# Dissecting the Autocrine and Paracrine Roles of the CCR2-CCL2 Axis in Tumor Survival and Angiogenesis

**DOI:** 10.1371/journal.pone.0028305

**Published:** 2012-01-18

**Authors:** Liat Izhak, Gizi Wildbaum, Steffen Jung, Avi Stein, Yuval Shaked, Nathan Karin

**Affiliations:** 1 Department of Immunology, Rappaport Institute for Medical Research, Bruce Rappaport Faculty of Medicine, Technion, Haifa, Israel; 2 Department of Pharmacology, Rappaport Institute for Medical Research, Bruce Rappaport Faculty of Medicine, Technion, Haifa, Israel; 3 Rappaport Institute for Medical Research, Bruce Rappaport Faculty of Medicine, Technion, Haifa, Israel; 4 Department of Urology Carmel Medical Center, Haifa, Israel; 5 Department of Immunology, The Weizmann Institute of Science, Rehovot, Israel; University of Nebraska Medical Center, United States of America

## Abstract

The CCL2 CCR2 axis is likely to contributes to the development and progression of cancer diseases by two major mechanisms; autocrine effect of CCL2 as a survival/growth factor for CCR2+ cancer cells and, the attraction of CCR2+ CX_3_CR1+tumor associated macrophages that in the absence of CCR2 hardly migrate. Thus far no in vivo system has been set up to differentiate the selective contribution of each of these features to cancer development. Here we employed a chimera animal model in which all non-malignant cells are CCR2−/−, but all cancer cells are CCR2+, combined with an adoptive transfer system of bone marrow (BM) CX_3_CR1+ cells from CCR2+ mice harboring a targeted replacement of the CX_3_CR1gene by an enhanced green fluorescent protein (EGFP) reporter gene (*cx_3_cr1* ^gfp^), together with the CD45.1 congene. Using this system we dissected the selective contribution of CX_3_CR1+CCR2+ cells, which comprise only about 7% of CD11b+ BM cells, to tumor development and angiogenesis. Showing that aside for their direct pro-angiogenic effect they are essential for the recruitment of other CD11b+ cells to the tumor site. We further show that the administration of CCR2-Ig, that selectively and specifically neutralize CCL2, to mice in which CCR2 is expressed only on tumor cells, further suppressed tumor development, implicating for the key role of this chemokine supporting tumor survival in an autocrine manner. This further emphasizes the important role of CCL2 as a target for therapy of cancer diseases.

## Introduction

Chemokines are small (∼8–14 kDa), structurally related proteins that regulate cell trafficking via interactions with a subset of seven-transmembrane, G protein-coupled receptors [1], [2], [3], [4]. Cancer cells express different chemokine receptors and also produce their ligands. One of them is CCR2 and its major ligand CCL2 (MCP-1). CCL2 is a major inflammatory chemokine that also participates in the regulation of cancer disease. CCL2 and other CCR2 ligands are highly expressed by various tumors, such as melanoma, breast cancer, ovarian cancer and cancer of the prostate (CaP) [5], [6], [7], [8], [9]. Their direct interaction is essential to support tumor survival and growth [10] by at list three complementary pathways: (1) An autocrine effect on malignant cells that produce CCL2, and other CCR2 ligands, and also express their receptor (CCR2) [10]; (2) Attraction of bone marrow derived monocytic cells from the bone marrow to target tissues by chemotaxis, and then inducing their differentiation into macrophages [11], in particular tumor associated macrophages (TAMs) [12], [13], [14], and (3) pro-angiogenic effects on the endothelium that also expresses CCR2 [15].

We have recently shown that CCL2 is predominantly expressed at the human primary tumor site of patients suffering from CaP, which leads to selective breakdown of immunological tolerance resulting in a production of anti-CCL2 autoantibodies that are likely to participate in the regulation of disease [16].

The current study focuses on differentiating the contribution of CCL2-CCR2 interaction within CCR2+ BM-derived CD11b+Gr1+ cells from the autocrine effect of CCL2 on CCR2 expressed by the cancer cells. For this purpose we elaborated a chimera system in which a Luciferase trafected CCR2+ cancer cell line (TRAMP-C1) is implanted into CCR2−/− mice that were then reconstituted with BM cells from CCR2+ immunocompetent mice. The expression of CCR2 displays plasticity during differentiation of BM monocytic and dendritic cells. A more stable biomarker for the precursors of these cells is the chemokine receptor CX_3_CR1 [17], [18], [19]. Hence, the current study uses an adoptive transfer system in which BM CX_3_CR1+ cells (*cx_3_cr1* ^gfp^) from CCR2+ mice harboring a targeted replacement of the CX_3_CR1gene by an enhanced green fluorescent protein (EGFP) reporter gene together with the CD45.1 are transferred into control CCR2−/− mice, to show that these cells, which are approximately 7% of BM CD11b+ cells, are not only essential for direct support of the tumor, but also obligatory for the recruitment of other BMD CD11b+ cells to support tumor development and angiogenesis.

Finally a soluble CCR2-Ig that selectively neutralizes CCL2 [20] was then used to determine the direct contribution of the autocrine interaction of CCL2-CCR2 on the cancer cells to tumor growth.

## Materials and Methods

### Animal Models

All animal work was conducted according to the Technion ethic committee guidlines. C57BL/6 (WT) were purchased from Harlan (Israel). Breeders of CD45.1+ and CCR2−/− C57BL/6 mice were purchased from The Jackson Laboratory (Bar Harbor, Maine). CX_3_CR1^gfp^ C57BL/6 mice harboring a targeted replacement of the cx 3 cr1 gene by an enhanced green fluorescent protein (EGFP) reporter gene [21], that were generated previously by one of us (SJ), have been crossed to mice bearing the CD45.1 allotype. All mice were maintained in IVC cages under pathogen-free conditions. At 6 weeks of age, mice were injected subcutaneously between the two flanks with 7×106 syngeneic TRAMP-C1 luc. were kindly provided by N. M. Greenberg [22]). Mice were monitored daily for evidence of illness. Tumor diameters were measured using a caliper. Tumor volume was calculated using the formula π/6×a×b2, where a is the longest dimension, and b is the width.

### CCR2-Ig soluble receptor

A soluble receptor encoding the E3 domain of CCR2 has been constructed as we described elsewhereb [20]. This soluble receptor effectively, and selectively neutralizes the in vivo activities of CCL2 and suppresses experimental autoimmune encephalomyelitis (EAE) and the growth of human prostate cancer cells in SCID mice CCR2-Ig [20].

### Micro-metastases Detection

Detection of micro-metastases was done according to [23], [24]. In a preliminary experiment in which we calibrated the metastatic tumor model, we observed that under our working conditions, i.v. injection of 5–7×10^6^tumor cells/mouse was essential to obtain clear micro-metastases at the bones and lungs. Due to the high number of injected cells (7×106 C-1.luc cells/mouse) these cells were administered at a relatively high volume of PBS (400 µl/mouse) in a protocol that included very slow administration up to 1 min.

Thirty days following reciprocal administration of 7×10^6^ tumor cells/mouse (s.c.) to form primary tumor, and the same number of cells administered i.v. to form micro-metastases tissues from brain, lungs, heart, liver, bones and the primary tumor sections were subjected to Luciferase immunohistochemical (IHC) detection, according [25], as follows: tissues were fixed in 10% buffered formalin, embedded in paraffin, and sectioned onto glass slides. Paraffin-embedded sections were hydrated through xylene and graded alcohol and equilibrated in PBS. Antigen retrieval was performed by heating the slides in 10 mM sodium citrate (pH 6.0) at 110°C for 4 min followed by staining for luciferase with monoclonal mouse anti-luciferase antibody (Clone LUC-1; Sigma) in a 1∶50 dilution. Staining was performed using EnVision Plus Systems (Dako) according to the manufacturer's protocol.

### Histology, Immunohistochemistry and Immunofluorescence

Samples were subjected to staining analysis [26] as follows: they were fixed in 10% formalin in PBS overnight, then dehydrated and embedded in paraffin. Immunohistochemistry analysis was performed on serial sections (5 µm) by an immunoperoxidase technique, using streptavidin-peroxidase and an AEC substrate chromogen kit (Zymed, San Francisco, CA). Next, tissue samples were deparaffinized, incubated with 3% H_2_O_2_ for 20 min to avoid endogenous peroxidase activity, and blocked using a non-immune blocking solution (Zymed) for 20 min. PBS-diluted primary antibodies were added and incubated overnight. After three washings, secondary antibody was applied for 1 h, followed by rinsing with PBS. After peroxidase staining, slides were counterstained lightly with hematoxylin. For F4/80+ cells we used a primary antibody polyclonal rat anti-mouse F4/80(MCA497B, Serotec, Raleigh, NC), for VEGF detection we used Polyclonal rabbit anti-VEGF (sc-152, Santa Cruze Biotechnology,) and for proliferating cells we used Mouse Anti mouse PCNA (MCA1558, Serotec, NC) [27]. Auto fluorescence staining of necrotic areas was done as we described in [28]. Anti CD31 staining was conducted on frozen sections using Rat anti Mouse CD31 mAb (PharMingen,) and FITC-goat anti-mouse IgG(Jackson ImmunoResearch,) according the protocol we described elsewhere [28].

### Imaging of Luciferase Activity In vivo

Imaging was performed using an IVIS200 Imaging System (Xenogen Biosciences). Mice were anesthetized using isoflurane and injected intraperitoneally with 150 mg/kg luciferin. Imaging was done 5 min after the luciferin injection. Quantification of light from specific regions was done using Living Image® software (Xenogen Corporation) and expressed as photons/second.

### Bone marrow–derived cell separation

Single-cell suspensions of bone marrow were prepared from the femurs of heterozygote mutant C57BL/6 mice (CD45^+^GFP^+^). Mononuclear cells were isolated using Lympholyte Separation Medium (Cedarlane Laboratories, Burlington, ON). CD11b+ cells were positively separated by cell separation kit (MACS beads, Miltenyi Biotec) and injected (i.v.) into CCR2−/− mice. GFP+ *(cx_3_cr1^gfp^*) cells were separated using FACSAria Cell-Sorting System (BD Biosciences, CA).

### Statistical analysis

The significance of differences was examined using Student's *t*-test. *P* values smaller than 0.05 were considered statistically significant.

## Results

### CCR2−/− mice display impaired development of CCR2+ primary tumors that become non-metastatic

**Figure 1:**
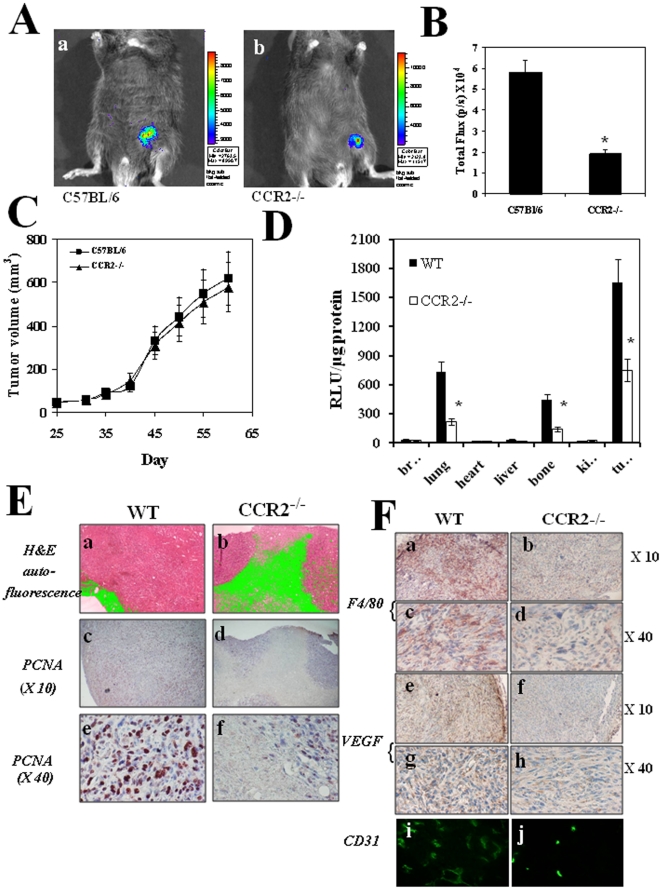
CCR2−/− mice display impaired development of CCR2+ primary tumors that become non-metastatic. (**A**) Six CCR2^+/+^ C57BL/6 mice (WT) and six CCR2^−/−^ C57BL/6 mice were administered with 7×10^6^ TRAMP C1-luc cells. Imaging of primary tumor was done on day 60, as recorded by the CCD camera (IVIS). Panels a & b show representative photos of CCR2^+/+^ C57BL/6 mice (WT) (a) and CCR2^−/−^ C57BL/6 mice (b) which were i.p injected with 200 µl luciferin 5 min before the exposure. (**B**) Computerized CCCD analysis of six mice per group. Results of six mice per group are shown as total flux (p/s ×10^4^) ±SE. * Indicates p<0.001. (**C**) Starting day 25, the two groups of mice were monitored for the development of the primary tumor. Results are shown as tumor volume ± SE. (**D**) Micro-metastases luminometer analysis of luc+ counts in organ sections obtained on day 50 from brain, heart, lungs, bones and primary tumor of CCR2+/+ C57BL/6 mice (WT) and CCR2−/− C57BL/6 mice administrated with 7×10^6^ C1-luc cells i.v, and the same number of cells s.c. to form primary tumor. Results are shown as mean relative light units per µg total protein, 9RLU/µg) ±SE. * Indicates p<0.001 (**E**) Histological and Immunohistochemical analyses of primary tumors from CCR2^+/+^ C57BL/6 mice (WT) and CCR2^−/−^ C57BL/6 mice. Panels a, b show H&E staining (×10) taken by fluorescence microscope, c–f show anti -PCNA staining; c, d (×10), e, f (×40). (**F**) Immunohistochemical and immunofluorescence analysis of primary tumors from CCR2^+/+^ C57BL/6 mice (WT) and CCR2^−/−^ C57BL/6 mice. Panels a–d show anti F4/80 staining; a, b (×10), c, d (×40) , e–h show anti VEGF staining; e, f (×10), g, h (×40) and i–j show anti CD31 staining (×40).

At the tumor site CCR2 is expressed on invading tumor cells, the endothelium, and TAMs. In an attempt to study the distinguishable contribution of non-malignant CCR2+ cells at the tumor site to tumor development and angiogenesis, wild-type and CCR2−/− C57BL/6 mice were administrated with 7×10^6^ CCR2+ TRAMP C1.luc cells that stably over-express a luciferase reporter gene. On day 65, when tumors reached 500–600 mm^3^, all mice were subjected to CCCD camera analysis, determining luciferase activity, which represents tumor cell viability and growth [29]. Figure 1A shows a CCCD camera photography of a representative control (CCR2+) mouse (a) compared to CCR2−/− mice (b), and analysis of mean total flux (photons per second) of all six mice within each experimental group (Fig. 1B). These results show a highly significant difference between both groups (5.81×10^4^±0.7 compared to 1.93×10^4^±0.09, p<0.001). Notably, the decreased luciferase activity in tumors implanted in CCR2−/− mice was not associated with a significant decrease in tumor size, as measured by caliper (Figure 1C).

We then monitored micro-metastases formation at the brain, liver, bones and lungs of CCR2−/− and WT mice subjected to i.v. + s.c administration of tumor cells. Fig. 1D shows analysis of the cellular localization and cell-specific staining for luciferase activity (relative light units per µg total protein, RLU/µg) of histological section of organs from all six mice. We could observe in WT mice an apparent RLU increase in lungs, bones and primary tumor, all of which were significantly decreased in CCR2−/− mice (730±80 compared to 220±30, 440±45 compared to 140±22, and 1650±180 compared to 760±80, p<0.001 for all 3 comparisons).

Taken together, these results show that CCR2−/− mice display a reduced development of the CCR2+ primary tumor, as determined by tumor cell viability and spread in various ograns, but this reduced enzymatic activity was not correlated with the primary tumor size. Of note, we have not yet ruled out the possibility that luciferase activity in tumors was reduced due to a reduction in angiogenesis, a point which we have further investigated as discussed below.

### Impaired development of CCR2+ primary tumor in CCR2−/− mice is associated with increased necrotic areas and reduced proliferative response localized within the central area of the tumor

To explain the discrepancy between the significant reduction in luciferase activity in primary tumors of CCR2−/− mice compared to wild type (Figure 1A and 1B), and the comparable tumor size between these groups, primary tumor sections from all mice of each group were subjected to histological and immunohistochemical analyses. The results in Figure 1E show representative sections (n = 18 per group) of harvested tumors. Auto fluorescence staining of necrotic areas [28] indicated large necrotic areas at the primary tumor site developed in CCR2−/− mice, when compared to wild-type counterparts (Figure 1E, b compared to a). In addition, PCNA staining for proliferating cells [27] revealed reduced proliferative responses in tumor sections obtained from CCR2−/− mice, when compared to their wild-type counterparts (Figure 1E d compared to c, and f compared to e). Notably, necrotic areas were localized within the central area of the tumor where cell proliferation was reduced, and not within the viable tumor rim. Collectively, these results suggest that reduced luciferase activity in tumors from CCR2−/− mice (Figure 1) resulted from impaired tumor proliferation followed by increased necrosis at the central areas of the tumor.

### Impaired development of CCR2+ primary tumor in CCR2−/− mice is associated with reduced accumulation of F4/80+ cells, reduced VEGF expression and lack of angiogenesis

CCL2 is an obligatory mediator in directing the mobilization of F4/80+ monocytes from the bone marrow via its interaction with CCR2 [30], [31]. Thus CCR2−/− mice are impaired in this feature, and its consequences [32], [33]. Further immuno-histological analysis of the sections showed that indeed CCR2−/− mice had a reduced number of F4/80^+^ cells at the tumor site (Figure 1F b compared to a and d compared to c). It should be noted that of the CD11b+ BM cells the monocytic cells (F4/80+), but not granulocytes are CCR2+. This receptor has been shown to be essential for monocytic exclusive exit from the BM [32], which is an essential step for their later accumulation at inflammatory/tumor sites. Here we show that in the absence of CCR2, F4/80+ cells also do not accumulate at the tumor site (Figure 1F).

The development and invasion of a tumor is largely dependent on its ability to induce angiogenesis, either by producing angiogenic factors, such as VEGF, in an autocrine manner, or indirectly via such factors produced by recruited BM cells or stroma cells at the tumor microenvironment. We therefore assessed the VEGF-A and CD31 (a marker for endothelial cell) expression in these tumor sections (Figure 1F) and found a markedly reduced staining of VEGF-A, that could explain, in part the significant reduction CD31+ endothelial cells in primary tumors from CCR2−/− when compared to tumors from WT mice.Collectively, the above observations suggest that F4/80+ tumor-associated macrophages are essential for sufficient production of angiogenic factors, including VEGF, at the tumor site. Thus their absence leads to impaired angiogenesis resulting in reduced proliferation and increased necrosis of tumor cells, particularly at the center of the tumor.

Collectively, the above observations suggest that the lack of both TAMs and angiogenesis in tumors grown in CCR−/− mice may account for the reduced tumor cell viability when compared to tumors grown in wild-type mice, but could not explain why tumor size remain similar in both groups.

### Bone marrow derived CD11b+CCR2+ cells are essential to support tumor development and angiogenesis

**Figure 2:**
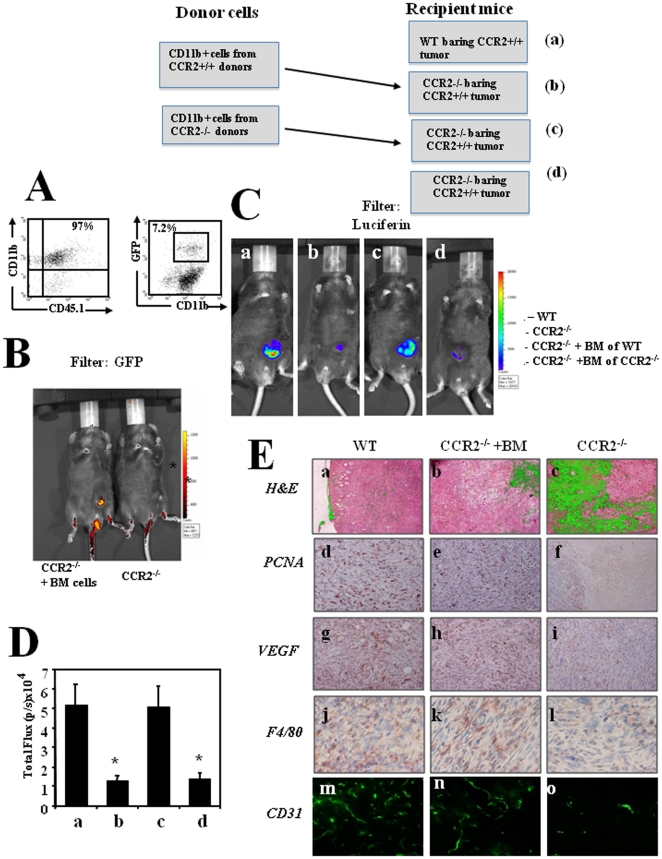
*Bone marrow derived CD11b+CCR2+ cells are essential to support tumor development and angiogenesis.* (**A**) CD11b+ BMD cells from *cx_3_cr1* ^gfp^ CCR2+ CD45.1 mice were purified (left panel), analyzed fro the relative mummer of GFP+ cells (right panel) and transferred to CCR2−/− mice bearing CCR2+ tumor (**B**) shows imaging (IVIS) of a representative mouse as recorded using a GFP filter. (**C**) Imaging (IVIS) of the primary tumor on day 60, as recorded by the IVIS camera using – luciferin filter (recording luciferase activity of the cancer cells) as follows: CCR2+/+ C57BL/6 mice (WT) (a), CCR2−/− mice (b), CCR2−/− transplanted with BM of WT mice(c) and CCR2−/− transplanted with BM of CCR2−/−mice. All photos show a representative mouse per group (1 of 6 mice). (**D**) The computerized CCCD analysis of six mice per group. Results are shown as total flux (p/s ×10^4^) ±SE. * Indicates p<0.001 (**E**) Histological, Immunohistochemical and immunofluorescence analyses of primary tumors from CCR2^+/+^ C57BL/6 mice (WT), CCR2^−/−^ C57BL/6 mice and BM transplanted CCR2^−/−^ mice. Panel a–c show H&E staining, d–f show anti -PCNA staining, g–i show anti F4/80, j–l show anti VEGF and m–o show anti CD31.

**Figure 3:**
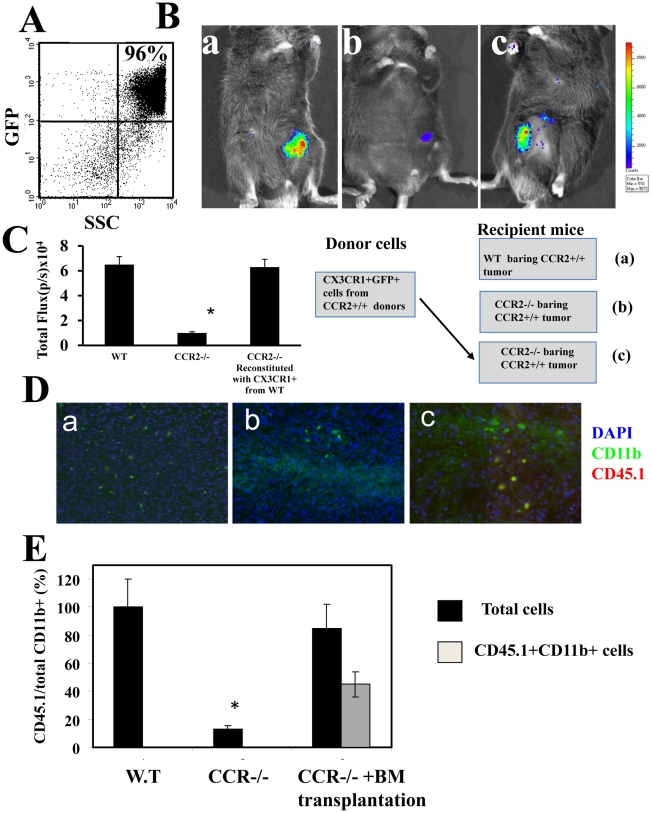
Bone marrow derived CX_3_CR1+ cells are drivers of tumor angiogenesis. (**A**) FACS analysis of *cx_3_cr1* ^gfp^ cells purified by FACSAria Cell-Sorting System from BM of CCR2+ CD45.1 donor mice before their transfer to CCR2−/− mice (**B**) Imaging (IVIS) of the primary tumor on day 60, as recorded by the IVIS camera using – luciferin filter (recording luciferase activity of the cancer cells) as follows: CCR2+/+ C57BL/6 mice (WT) (a), CCR2−/− mice (b), CCR2−/− transplanted with GFP+ cells from BM of CCR2+ donor mice (c). All photos show a representative mouse per group (1 of 6 mice). (**C**) Computerized CCCD analysis of six mice per group. Results of six mice per group are shown as total flux (p/s ×10^4^) ±SE. * Indicates p<0.001. (**D**) Representative primary tumor sections were then analyzed by to immunostaining using different colors for CD45.1 (red color, only transferred *cx_3_cr1* ^gfp^ cells) and CD11b+ (green). (**E**) Analysis of 60 sections from six mice per group for the relative number of CD11b+ cells at tumor sections from each group, and of CD45.1 cells following cell transfer * Indicates p<0.001.

In an attempt to delineate the role of BM CCR2+ DC/monocytic cells to tumor development we have conducted two complementary sets of adoptive transfer experiments. The first experiment compared the ability of either BM CD11b+ cells from CCR2+ donors to support tumor development in CCR2−/− mice (Fig. 2), and the other experiment compared the ability of purified CX_3_CR1 (GFP+) cells from CCR2+ mice to do so (Fig. 3). In both experimental systems BM donor CCR2+ cells were isolated from CD45.1mice harboring a targeted replacement of the CX_3_CR1gene by EGFP (*cx_3_cr1* ^gfp^). At first, total CD11b+ cells were isolated (Fig. 2A 97% purity). Within this CD11b+ population the *cx_3_cr1* ^gfp^ cells were as few as 7.2% (Fig. 2A). The CD11b+ cells from these donors were administered (5×10^6^/mouse) one week before the subcutaneous implementation of 7×10^6^ CCR2+ TRAMP C1.luc cells. On day 65, when tumors reached a size of 500–600 mm^3^, all mice were subjected to CCCD camera analysis. Fig. 2B shows that the BM *cx_3_cr1* ^gfp^ cells from CCR2+ donors selectively accumulated at the tumor site. Fig. 2C follows the luciferase activity, (representing tumor cell viability) of the TRAMP C1.luc cells in representative mice from groups of either WT or CCR2−/− mice, that were, or were not reconstituted with the CD11b+ cells from either CCR2+/+ or CCR2−/− mice. These results represent one of three different experiments, each with 6 mice per group. In all experiments we observed that BMcells from CCR2+/+ mice, but not from CCR2−/− donors could restore tumor growth to comparable levels that were found in tumors from WT mice. Fig. 2D shows analysis of the mean total flux (photons per second) of all six mice within each experimental group showing that the decrease in total flux observed in CCR2−/− (b compared to a, 1.63±0.3×10^4^ compared to 5.20±1.1×10^4^, p<0.001) could be fully restored by BM cells from CCR2 WT but not from BM cells from CCR2−/− mice (d compared to c, 1.7±0.25 compared to 5.11±0.95, p<0.001).

Histological and immunohistological analyses of representative sections from each group (18 sections from six mice per group) show a significant reduction of necrotic areas (auto-fluorescence staining) in tumors from CCR2−/− mice administered with CD11b+ cells from CCR2+/+ donors (Figure 2E b compared to c) accompanied by increased PCNA staining representing proliferating cells (Figure 2E f compared to e). Furthermore, an increased VEGF expression (Figure 2E h compared to i) as well as accumulation of F4/80+ TAMs (Figure 2E k compared to l) were also observed in tumors from CCR2−/− mice administered with CD11b+ cells from CCR2+/+ donors. To further assess whether reduced expression of VEGF (and possibly other proangiogenic factors) at the tumor site accompanied with reduced formation of blood vessels, primary tumor sections from all groups were subjected to immunostaining with anti-CD31 antibodies (a marker of endothelial cell). Fig. 2E clearly shows a markedly reduced staining of CD31+ endothelial cells in primary tumors from CCR2−/− mice when compared to tumors from WT mice (Figure 2E o compared to m). It should be noted that CD31 is also expressed on macrophages [34], however Figure 2 clearly show a marked reduction in staining that well characterizes vessel structure. These results indicate that the reducedangiogenesis in such tumors (see also Fig. 1), could be restored following BM CD11b+CCR2+ cell transplantation (Figure 2E n).

Collectively, these results suggest that BM CD11b+CCR2+ cells home via CCR2 to the tumor to support its angiogenesis and subsequent growth by eliciting the production of angiogenic factors and possibly by other mechanisms, yet to be identified.

### Bone marrow derived CX_3_CR1+ cells are drivers of tumor angiogenesis

The CX_3_CR1+ cells (in our system, *cx_3_cr1* ^gfp^) are approximately 7% of CD11b+ BM cells (Fig. 2A). To assess their contribution to tumor development, we have purified them in two steps. At first, total CD11b+ BM donor CCR2+ cells were isolated from CD45.1 mice, harboring a targeted replacement of the CX_3_CR1gene by EGFP (*cx_3_cr1* ^gfp^). Then GFP+ cells were isolated using FACSAria Cell-Sorting System (Fig. 3A, 96% purity), and administered (3×10^6^/mouse) to CCR2−/− mice bearing a CCR2+ tumor, under the same experimental conditions as described above (Fig. 2). Fig. 3B shows the results obtained from representative mice (CCCD camera) whereas Figure 3C sumerizes the mean total flux of six mice per group. Together they show that administration of CX3CR1+GFP cells from CCR2+/+ mice to CCR2−/− mice barring a CCR2+/+ tumor could fully restore tumor development in CCR2−/− mice (Fig. 3C, 6.5±0.5 in WT compared to 1.2±0.3 in CCR2−/−, p<0.001, and 6.3±0.6 in BM cells reconstituted mice). Primary tumor sections were then analyzed by immunostaining using different chloroforms for CD45.1 (only transferred *cx_3_cr1* ^gfp^ cells) and CD11b+ (Fig. 3D). Analysis of 60 sections from six mice per group (Fig. 3E) revealed that: a. In the absence of *cx3cr1* ^gfp^ cells from CCR2+ mice, endogenous CD11b+ cells hardly accumulated at the tumor site (about 20 folds less than tumors from WT mice) b. Accumulation of endogenous CCR2−/− CD11b+ cells was reconstituted following the administration of *cx3cr1* ^gfp^ cells almost to the same levels found in tumors of WT mice (approximately 85%) to support tumor angiogenesis. Taken together, our results suggest that BM CCR2+ CX_3_CR1 cells are drivers of tumor support not only due to their potential direct function, but also due to their ability to recruit other CCR2−/− BM cells to the tumor site.

### The direct effect of CCL2 on the in vivo development and progression of the primary tumor

**Figure 4:**
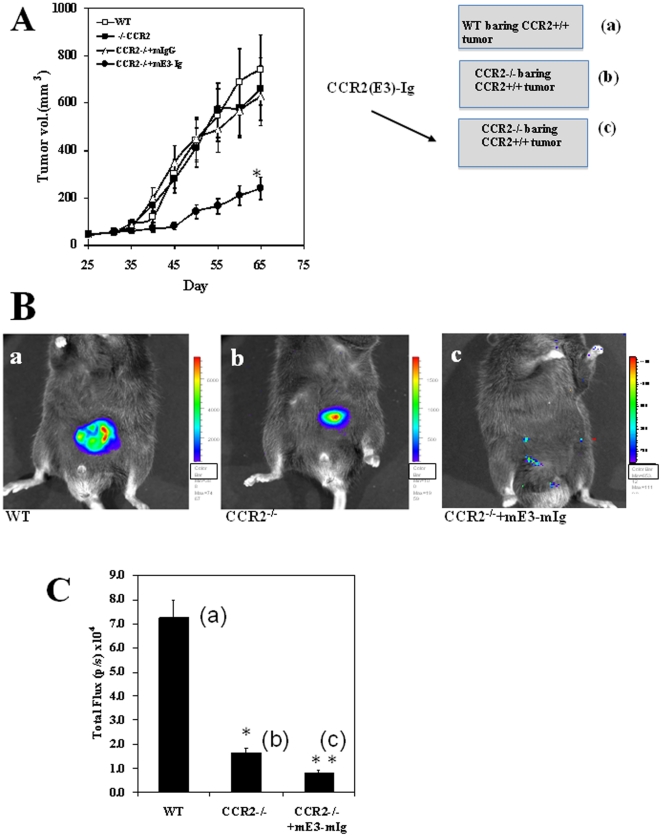
mE3-mIg inhibits the development of primary tumor in CCR2^−/−^ mice. (A) Three groups of CCR2^−/−^ and one of C57BL/6 mice were administered 7×10^6^TRAMP C1-luc cells. 25 days later, mice were repeatedly administered (every 3 days) with 200 µg mE3-Ig, isotype-matched control mIgG or PBS and monitored for the development of the primary tumor. Results are shown as tumor volume ± SE. * Indicates p<0.001. (B) Imaging of the primary tumor was done on day 65, as recorded by the CCD camera(IVIS).Panels a, b & c show representative photos of a CCR2^+/+^ C57BL/6 mouse (a), CCR2^−/−^ C57BL/6 mouse (b) and CCR2−/− mouse treated with mE3-mIg (c) which were i.p injected with 200 µl luciferin 5 min before the exposure . (C) Summery of the computerized CCCD analysis of six mice per group of control mice (WT), CCR2^−/−^ mice and those treated with mE3-mIg. Results are shown as total flux (p/s ×10^4^) ±SE. * Indicates p<0.001 when comparing b and c to a, ** Indicates p<0.001 p<0.005 c to b.

The next approach was to use our experimental system to delineate the direct contribution of the interaction between the CCR2+ tumor cells and the CCL2 ligand on tumor growth. The basic experimental system is identical to the one we used above in which only the tumor cells expressed CCR2. Here we have added an additional group in which the CCR2−/− mice that were implanted with the Luc+ CCR2+ tumor cells (C1-TRAMP) were subsequently injected (beginning 25 days after tumor injection, every 5 days, 300 µg/mouse) with a soluble CCR2-Ig receptor that effectively and selectively neutralizes only CCL2 [20]. In accordance with the results shown in Figure 1 CCR2−/− mice bearing CCR2+/+ tumor cells did not differ in tumor size from control WT mice (Fig. 4A), yet they displayed a significantly decreased luciferase activity (Figure 4B, C, 7.31±1.4 compared to 1.69±0.35, p<0.01). However, the repeated treatment with of the CCR2-Ig not only further suppressed luciferase activity (Figure 4B, C, p<0.01), but also led to a significant inhibition in tumor size (Figure 4A, day 65, 227±19 compared to 770±65 mm^3^, p<0.001). These results implicate an important role of CCL2 in supporting tumor development in an autocrine manner.

## Discussion

There is a growing interest in studying the role of BM cells in tumors, in part due to the presence of ‘accessory’ BM stroma cells which have been shown to contribute to tumor angiogenesis, tumor cell repopulation, metastatic spread and growth, as well as in cases where tumors acquire resistance to antiangiogenic therapy [35], [36], [37], [38]. Studies using Gr1+/CD11b+ cells have recently shown to reduce the efficacy of anti-angiogenic therapy neutralizing the VEGF-A activity [38]. These cells home to treated tumor sites and release a number of proangiogenic factors that compensate on the lack of VEGF [38].

One of the intriguing observations of the current manuscript is that in the growth kinetics of CCR2+ tumor implanted in CCR2−/− mice is comparable with the one developed in CCR2+ mice (Fig. 1C), whereas there is a huge reduction in illumination as observed by CCCD camera (Fig. 1A, B). Fig. 1E, F provides some explanation to this discrepancy. It shows, by auto fluorescence staining, large necrotic areas at the primary tumor site developed in CCR2−/− mice, when compared to wild-type mice (Fig. 1E, b compared to a), together with a marked reduction in F4/80 cell accumulation at this site (Fig. 1F, b compared to a and d compared to c). Together with the complementary experiment showing that reconstitution of CCR2−/− mice with CX3CR1+CCR2+ BM cells from WT mice overcomes the reduction of fluorescence emission of this tumor (Fig. 3B) our study implies that in the absence of CCR2+ BM derived cells the tumor, even though displays growth kinetics that is comparable with tumors of wild-type mice, yet it undergoes necrosis. This further emphasizes the role of CCR2+ BM derived cells in tumor development and survival.

Bone Marrow CD11b+Gr1+ cells, include neutrophils, monocytic cells, and myeloid-derived suppressor cells, all of which are likely to contribute to tumor angiogenesis [28], [38], [39], [40], [41]. Of these cells, only the monocytic cells and the monocytic derived DC express CCR2. For these cells the interaction of CCR2+ and its ligands is essential for directing their mobilization from the BM [30], [31]. Thus CCR2−/− mice are impaired in this feature, and its consequences as partially revealed by this study [33]. In an attempt to delineate the contribution of these cells to the pathogenesis of prostate cancer, we used a chimera system in which the Luc+ CCR2+ tumor cells (C1-TRAMP) were administered into CCR2−/− immunocompetent mice, which subsequently, were, or were not reconstituted with CCR2+ CD11b+ BM cells, or with CCR2−/− CD11b+ BM cells. We showed that only BM cells from CCR2+ mice could fully restore tumor development, implicating for the pivotal role of the CCR2+ BM cells in supporting tumor development and angiogenesis (Fig. 2).

At the tumor site, the vast majority CCR2+ cells are TAMs. Previous studies have indicated that TAMs display a profound influence on the regulation of tumor angiogenesis, including promoting microvessel sprouting (reviewed in [42] and [41]). In a transgenic mouse mammary tumor model that expresses Polyoma Middle T antigen (MMTV-PyMT), the depletion of macrophages led to a marked reduction in vascular density, causing delayed tumor progression and metastasis. Reintroduction of macrophages into these mice led to a significant increase in vascular density and enhanced tumor progression [43]. Very recently Qian et al elegantly demonstrated the pivotal role of CCL2 in recruiting TAMS to support breast tumor metastasis [44]. It is likely that these TAMs are more essential at early stages of tumor angiogenesis, particularly for tumors that produce limited levels of angiogenic factors that would be sufficient for their angiogenesis. It is intriguing, however, that even BM CCR2+ cells (macrophages and DC) are only a small portion of BM cells that home and produce cytokines, chemokines, and angiogenic factors at the tumor site, and yet their role in such tumors is so essential. After all, BM CCR2 monocytic cells, neutrophils, and myeloid-derived suppressor cells also contribute to tumor development and progression by producing VEGF and other angiogenic factors, and they are the vast majority of CD11b+ cells that home to the tumor site. If so, why is the absence of CCR2+ cells so critical for tumor support? To address this question we have generated an adoptive transfer system in which purified BM GFP+ cells from CCR2+ mice harboring a targeted replacement of the CX_3_CR1gene by EGFP reporter gene (*cx_3_cr1* ^gfp^), together with the CD45.1 congene, were transplanted into CCR2−/− mice bearing a CCR2+ tumor. We showed that these cells not only exclusively accumulated at the tumor site (Fig. 2B), but they were also essential for the recruitment of other BM cells and therefore are likely to support tumor growth (Fig. 3).

We therefore suggest a multi-step model in which CCR2+ CD11b+ BM cells accumulate at the tumor site to initially support tumor development and angiogenesis, resulting in enhanced levels of various inflammatory cytokines, growth factors and chemokines, that enable the rapid attraction of other BM cells, including those that are CCR2−, to further assist tumor development and progression. CCR2 is expressed on two different types of none-malignant cells at the tumor site: the endothelium [15], and the TAMs. Our results showing that BM reconstitution of CCR2+ cells in CCR2−/− mice bearing a CCR2+ tumor fully reconstitutes its development; therefore support the pivotal role of these receptors on BM cells in supporting tumor angiogenesis.

It is likely that most of contribution of the BM transferred cells from CCR2+ to CCR2−/− mice resulted from the ability of CCR2+ monocytic cells to directly support the tumor, and to recruit other BM cells. Yet, it should be noted, that potentially some BM cells transferred, could re-differentiate into mature endothelial cells [45], and by that, they can override the absence of CCR2 on endothelial cells of the CCR2−/− BM recipient. Using a direct immunostaining of CCR2 on histological sections from mice transferred with *cx_3_cr1^gfp^* cells we could not identify CCR2+ endothelial cells (data not shown).

Our system also enabled determining the direct autocrine effect of the CCL2-CCR2 autocrine interaction when CCL2 binds its CCR2 receptor on the tumor cells. Several ligands bind CCR2, yet CCR2 is likely to be the exclusive target of CCL2. We show that the administration of CCR2-Ig that selectively neutralize CCL2 [20] entirely suppress tumor development in CCR2−/− mice, where the only cells that were CCR2+ were the tumor cells (Fig. 4). This distinguishes, for the first time the autocrine from the paracrine effect of this key chemokine in the regulation of cancer diseases.
