# Correction: Dissecting the Autocrine and Paracrine Roles of the CCR2-CCL2 Axis in Tumor Survival and Angiogenesis

**DOI:** 10.1371/journal.pone.0195170

**Published:** 2018-04-12

**Authors:** Liat Izhak, Gizi Wildbaum, Steffen Jung, Avi Stein, Yuval Shaked, Nathan Karin

In Fig 2E, the wrong image appears in panel j and there are errors in the associated caption for panels g-i and j-l. Please see the corrected Fig 2 here.

**Fig 2:**
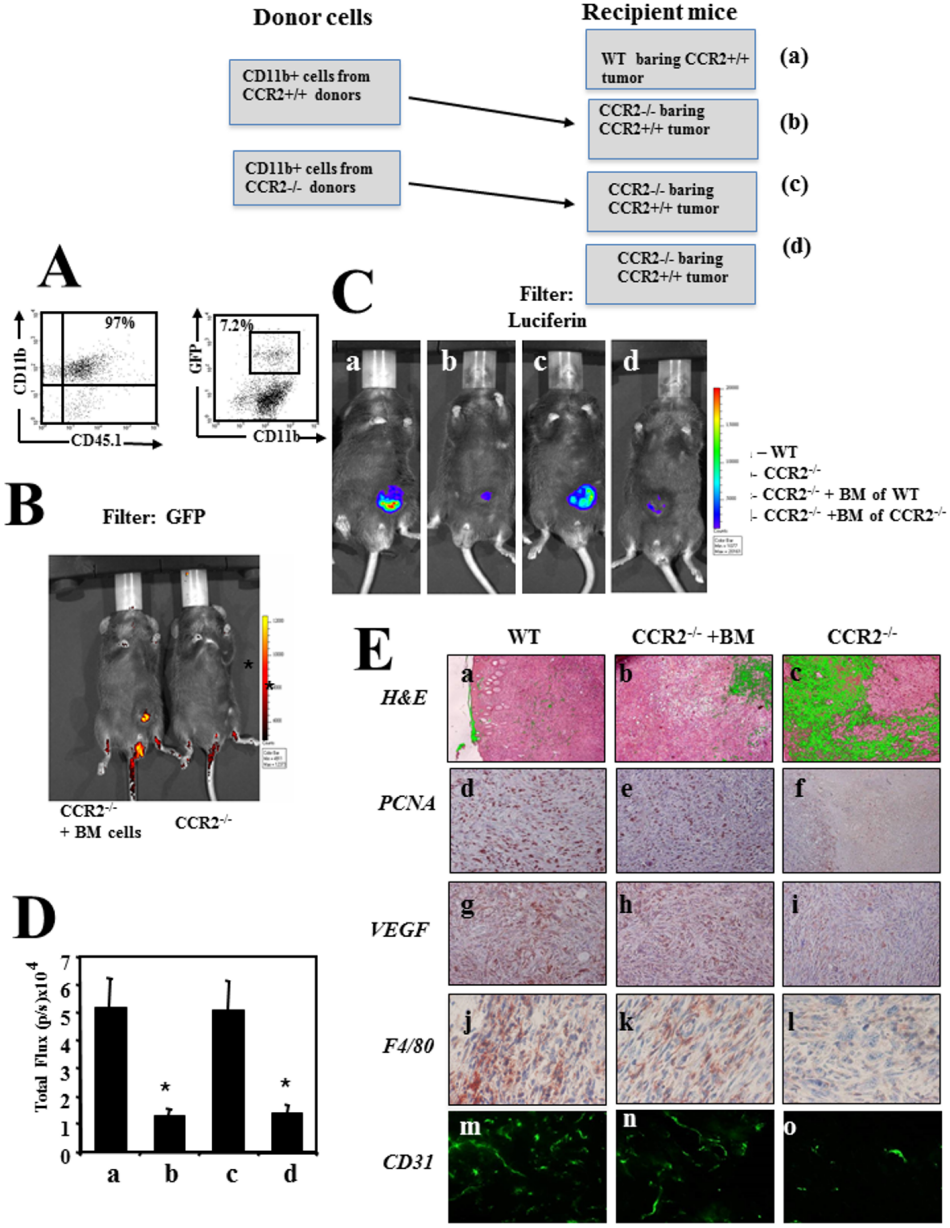
Bone marrow derived CD11b+CCR2+ cells are essential to support tumor development and angiogenesis. (**A**) CD11b+ BMD cells from cx3cr1gfp CCR2+ CD45.1 mice were purified (left panel), analyzed fro the relative mummer of GFP+ cells (right panel) and transferred to CCR2−/− mice bearing CCR2+ tumor (**B**) shows imaging (IVIS) of a representative mouse as recorded using a GFP filter. (**C**) Imaging (IVIS) of the primary tumor on day 60, as recorded by the IVIS camera using–luciferin filter (recording luciferase activity of the cancer cells) as follows: CCR2+/+ C57BL/6 mice (WT) (a), CCR2−/− mice (b), CCR2−/− transplanted with BM of WT mice(c) and CCR2−/− transplanted with BM of CCR2−/−mice. All photos show a representative mouse per group (1 of 6 mice). (**D**) The computerized CCCD analysis of six mice per group. Results are shown as total flux (p/s ×104) ±SE. * Indicates p<0.001 (**E**) Histological, Immunohistochemical and immunofluorescence analyses of primary tumors from C57BL/6 WT mice, CCR2-/- (KO) mice, and CCR2-/- (KO) mice reconstituted with BM from WT mice. Panel a-c show H&E staining, d-f show anti -PCNA staining, g-i show anti VEGF, j-l show anti F4/80, m-o show anti CD31.
